# Spinal Cord Excitability and Sprint Performance Are Enhanced by Sensory Stimulation During Cycling

**DOI:** 10.3389/fnhum.2017.00612

**Published:** 2017-12-18

**Authors:** Gregory E. P. Pearcey, Steven A. Noble, Bridget Munro, E. Paul Zehr

**Affiliations:** ^1^Rehabilitation Neuroscience Laboratory, University of Victoria, Victoria, BC, Canada; ^2^Human Discovery Science, International Collaboration on Repair Discoveries (ICORD), Vancouver, BC, Canada; ^3^Centre for Biomedical Research, University of Victoria, Victoria, BC, Canada; ^4^Nike Exploration Team Sport Research Laboratory, Nike Inc., Beaverton, OR, United States; ^5^Division of Medical Sciences, University of Victoria, Victoria, BC, Canada

**Keywords:** H-reflex, sensory stimulation, fatigue, spinal cord excitability, sprints, cycling

## Abstract

Spinal cord excitability, as assessed by modulation of Hoffmann (H-) reflexes, is reduced with fatiguing isometric contractions. Furthermore, spinal cord excitability is reduced during non-fatiguing arm and leg cycling. Presynaptic inhibition of Ia terminals is believed to contribute to this suppression of spinal cord excitability. Electrical stimulation to cutaneous nerves reduces Ia presynaptic inhibition, which facilitates spinal cord excitability, and this facilitation is present during arm cycling. Although it has been suggested that reducing presynaptic inhibition may prolong fatiguing contractions, it is unknown whether sensory stimulation can alter the effects of fatiguing exercise on performance or spinal cord excitability. Thus, the aim of this experiment was to determine if sensory stimulation can interfere with fatigue-related suppression of spinal cord excitability, and alter fatigue rates during cycling sprints. Thirteen participants randomly performed three experimental sessions that included: unloaded cycling with sensory stimulation (*CONTROL + STIM*), sprints with sensory stimulation (*SPRINT + STIM*) and sprints without stimulation (*SPRINT*). Seven participants also performed a fourth session (*CONTROL*), which consisted of unloaded cycling. During SPRINT and SPRINT + STIM, participants performed seven, 10 s cycling sprints interleaved with 3 min rest. For *CONTROL* and *CONTROL + STIM*, participants performed unloaded cycling for ~30 min. During *SPRINT + STIM* and *CONTROL + STIM*, participants received patterned sensory stimulation to nerves of the right foot. H-reflexes and M-waves of the right soleus were evoked by stimulation of the tibial nerve at multiple time points throughout exercise. Sensory stimulation facilitated soleus H-reflexes during unloaded cycling, whereas sprints suppressed soleus H-reflexes. While receiving sensory stimulation, there was less suppression of soleus H-reflexes and slowed reduction in average power output, compared to sprints without stimulation. These results demonstrate that sensory stimulation can substantially mitigate the fatiguing effects of sprints.

## Introduction

It is well established that intermittent, maximal effort bouts of exercise result in decrements in performance. In many cases, these performance decrements are due to neuromuscular fatigue that is a combination of peripheral and central fatigue mechanisms (Billaut et al., 2006; Billaut and Basset, 2007; Racinais et al., 2007a; Mendez-Villanueva et al., 2008; Girard et al., 2013a,b; Pearcey et al., 2015b, 2016; Monks et al., 2017). Although not the only mechanism, central fatigue resulting from high intensity cycling (Amann, 2012; Amann et al., 2013; Sidhu et al., 2014, 2017) is believed to result from group III/IV muscle afferent feedback, which subsequently alters corticospinal excitability and the level of muscle activation (Pearcey et al., 2016). In felines, fatiguing stimulation of the gastrocnemius-soleus muscles results in suppression of monosynaptic Ia activation of synergistic motoneurones, which is primarily due to increased pre-synaptic inhibition (Kalezic et al., 2004). Furthermore, fatiguing voluntary (Kukulka et al., 1986; Iguchi and Shields, 2012), non-voluntary electrically-evoked (Garland and McComas, 1990) and sub-maximal voluntary (Kuchinad et al., 2004) contractions of the plantarflexors, and voluntary contractions of the intrinsic hand muscles (Duchateau and Hainaut, 1993; Duchateau et al., 2002) cause suppression of Hoffmann (H)-reflex amplitudes. Prolonged running also causes drastic suppression of the Soleus (SOL) H-reflex for at least 30 min (Racinais et al., 2007b). Although it seems likely that H-reflex amplitudes would be reduced, it is currently unknown whether cycling sprints affect spinal reflex excitability in humans.

Although the effects of sprint cycling on H-reflex amplitudes are not known, non-fatiguing arm (Frigon et al., 2004; Loadman and Zehr, 2007; Barzi and Zehr, 2008; Hundza and Zehr, 2009; de Ruiter et al., 2010; Palomino et al., 2011) and leg (Motl and Dishman, 2003; Motl et al., 2003, 2004, 2006) cycling effects on H-reflexes have been studied extensively. With both paradigms, there is suppression of SOL H-reflex amplitudes. Cycling-induced suppression has been attributed to increased presynaptic inhibition from Ia presynaptic inhibitory interneurons (Brooke et al., 1997; Frigon et al., 2004). Since the available evidence suggests that fatiguing isometric contractions and non-fatiguing cycling both cause suppression of the SOL H-reflex amplitude, it seems likely that fatiguing cycling would do the same.

The importance of afferent input to motoneurons during voluntary contractions was demonstrated by Macefield et al. (1993). They showed that firing rates of the tibialis anterior (TA) are reduced when deprived of afferent feedback from the contracting muscle. More recently, Baudry et al. (2011) have shown that reductions in presynaptic inhibition of Ia afferents are associated with an increased time that a fatiguing contraction can be sustained. One method known to reduce presynaptic inhibition of Ia afferents is electrical stimulation of cutaneous afferents (Hagbarth, 1952). Electrical stimulation of nerves innervating the skin of the hand (Zehr et al., 2004) and foot (Demairé et al., 1989; Iles, 1996; Brooke et al., 1997; Frigon et al., 2004) can decrease Ia presynaptic inhibition and, therefore, increase the amplitude of H-reflexes. Furthermore, cutaneous electrical stimulation applied during and after cycling has been shown to “cancel” the inhibitory effects of cycling on H-reflex amplitudes (Frigon et al., 2004; Javan and Zehr, 2008). Therefore, since the facilitation of sensory stimulation and fatiguing effects of exercise on the H-reflex both act, at least partially, via Ia inhibitory mechanisms, the combination of sensory stimulation during fatiguing cycling may offset one another, resulting in less decrement of spinal excitability and cycling performance.

Thus, the purpose of the current study was to: (1) confirm that fatigue via intermittent sprints will decrease spinal reflex excitability; (2) determine whether patterned sensory stimulation can increase spinal reflex excitability during unloaded cycling; and (3) determine if patterned sensory stimulation can interact with fatigue to mitigate suppression of reflexes and decrements in performance. Based on previous fatiguing and non-fatiguing cycling literature, we hypothesize that H-reflex amplitudes would decrease as a result of the fatigue induced from intermittent sprints. Furthermore, due to the fact that sensory stimulation reduces pre-synaptic inhibition of Ia presynaptic inhibitory interneurons, we hypothesize that patterned sensory stimulation would increase H-reflex amplitudes during unloaded cycling. Lastly, we hypothesize that patterned sensory stimulation would have an interaction effect during cycling that would decrease the rate of power decrement and offset the suppression of H-reflexes due to fatigue.

## Materials and Methods

### Participants

Thirteen volunteers (24.3 ± 3.10 years, 174.3 ± 7.43 cm, 74.5 ± 12.03 kg) of both sexes (3 female) participated in this experiment. Participants had no known history of metabolic or neuromuscular impairment. Participants provided written and signed informed consent. The study was conducted in accordance with the Human Research Ethics Board at the University of Victoria (UVic) and the Declaration of Helsinki. The protocol was approved by the UVic Human Research Ethics Board.

### Experimental Protocols

In order to examine the effects of sensory stimulation of the right foot on fatigue and H-reflexes during fatiguing cycling, the following three experimental protocols were used: (1) *No Sprint (CONTROL + STIM)*; (2) *No Stim Sprint (SPRINT)*; and (3) *Stim Sprint (SPRINT + STIM)*. All three protocols were randomly performed at the same time of day (± 60 min) with a minimum of 48 h (up to 7 days) between protocols. To ensure there were no affects of the unloaded cycling used in the outlined experiments on H-reflex amplitudes, seven participants performed a fourth condition (*CONTROL*). Participants were instructed to wear the same clothing and shoes for all experimental testing and asked not to perform heavy exercise, eat, drink caffeine, smoke, or drink alcohol 4 h prior to arriving at the laboratory.

**Figure 1:**
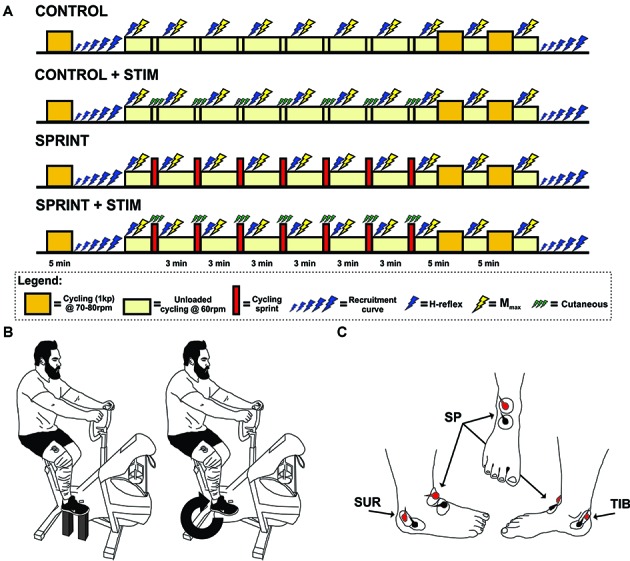
**(A)** Timeline of each experimental session. The legend below the timelines outlines the symbol meanings. **(B)** An illustration of the experimental set-up. The left portion of the illustration shows the position of the pre and post session M-H recruitment curve set-up for each participant. The right portion shows the position of H-reflex and *M*_max_ stimuli during cycling. This position was also the position that the train of sensory stimulation was evoked during the *SPRINT + STIM* and *CONTROL + STIM* conditions. **(C)** An illustration of the approximate electrode positions on the right foot for sensory stimulation. Views are of the lateral, frontal and medial from left to right, respectively.

Upon arrival, participants were informed of the protocol. The duration of each experimental testing session was the same for all four protocols, but the set-up time was slightly shorter for the *SPRINT* and* CONTROL* protocols because they did not require set-up of sensory stimulation of the foot. For all reflex measures, each participant’s posture was tightly monitored and controlled. That is, the seat height was adjusted to the comfort of the participant, knowing that they were to remain seated throughout the entire experiment. The selected seat height was then kept constant between all four protocols. Participants’ feet were secured on the pedals with nylon straps, and not altered throughout the session. Furthermore, during times that reflexes were recorded, the participants were instructed to hold the handlebars in a predetermined location with elbows and shoulders extended and asked to stare at a predetermined target directly in front of them (see Figure 1B for position). Following set-up, participants performed a standard warm-up of 5 min cycling at 70–80 rpm with a resistance of 1 kg. The participants then rested while resting M-H recruitment curves were performed. Finally, 10 H-reflexes with constant M-wave amplitude and three *M*_max_ were collected during unloaded cycling at 60 rpm and averaged.

The participants then either performed a 10 s sprint without any sensory stimulation (*SPRINT*), a sprint with sensory stimulation (*SPRINT + STIM*), continued to cycle (unloaded) at 60 rpm for 10 s while receiving sensory stimulation (*CONTROL + STIM*), or continued to cycle (unloaded) at 60 rpm for 10 s without stimulation (*CONTROL*). Participants then immediately returned to 3 min of cycling (unloaded) at 60 rpm while H-reflexes (10 sweeps) and *M*_max_ (3 sweeps) were evoked. During the evoked H-reflexes and *M*_max_ trials, participants were instructed to maintain the same posture as the pre-exercise test. When recording was complete, they were asked to cycle at 60 rpm and were free to move their upper body and talk until the next sprint (*SPRINT* and *SPRINT + STIM*), 10 s of sensory stimulation (*CONTROL + STIM*), or 10 s of unloaded cycling (*CONTROL*). The participants then repeated this procedure for a total of seven times. After the 7th repetition of this procedure, participants performed a 10 min cool-down by cycling at 70–80 rpm with 1 kp of resistance. *M*_max_ and H-reflexes were evoked at the 5 and 10 min marks (participants reverted back to 60 rpm unloaded cycling for collection). After the final H-reflexes and *M*_max_ were recorded, M-H recruitment curves were recorded at rest in the same position as pre-exercise (see Figure 1A for a timeline of each session).

### General Experimental Arrangements

#### Electrical Nerve Stimulation

##### Soleus H-reflex stimulation

To evoke H-reflexes in the SOL muscle, single 1 ms square wave electrical pulses were applied to the right popliteal fossa with bipolar surface electrodes (Thought Technology Ltd., Montreal, QC, Canada) using a Digitimer (Medtel, NSW, Australia) constant current stimulator (model DS7A). A non-contact milliammeter (mA-2000, Bell Technologies, Orlando, FL, USA) was used to measure current delivered for each stimulus. Recruitment curves (40 sweeps) were recorded at rest pre- and post-exercise. H-reflexes with an amplitude corresponding to ~75% *H*_max_ on the ascending limb of the recruitment curve with constant and measureable M-wave amplitude were recorded during unloaded cycling throughout each experimental session. This amplitude was chosen to ensure we were within the range of H-reflex amplitudes that are sensitive to both facilitation and inhibition from presynaptic inhibitory input (Crone et al., 1990). Furthermore, it was essential that there was a measureable M-wave amplitude to control the level of motoneuron excitability throughout the experiments (Zehr, 2002).

##### Stimulus-response curves

At rest, participants had their right foot at the 2 o’clock position of the cycle ergometer, secured with wooden blocks under the pedals and heel of the right foot (see Figure 1B for set-up). Knee and ankle angles (knee ~90°, knee ~120°) were monitored by the same investigator with a manual goniometer and kept constant from pre- to post-exercise and from session to session. Stimulus-response curve stimuli were delivered pseudo randomly between 1 s and 3 s for a total of 40 sweeps. Stimulus intensity was increased and decreased incrementally (ranged from 0.1 mA to 1 mA per increment) based on the excitability of the reflex pathway in different individuals, while ensuring that supramaximal M-wave amplitudes were achieved by increasing larger increments once the H-reflex amplitude started to decrease in size (i.e., after the ascending limb, for examples see Klimstra and Zehr, 2008).

##### Constant M-wave and H-reflex stimuli during cycling

To monitor the H-reflex excitability throughout the experiment, the current required to evoke a constant M-wave that corresponded to ~5% of *M*_max_ was delivered randomly every 1–2 pedal strokes at the 2 o’clock position during unloaded cycling (60 rpm). Pre-exercise H-reflex amplitudes at this stimulation intensity evoked a response that was ~75% of *H*_max_. A total of 10 sweeps were recorded, averaged and compared at each time point.

##### Supramaximal M-wave stimuli during cycling

To account for any peripheral changes in excitability throughout the experimental sessions, three supramaximal M-wave (*M*_max_) stimuli were delivered immediately after recording the 10 H-reflexes but at the same point in the cycle as the H-reflexes. Stimulation intensity was set to 140% of the value that produced *M*_max_ during the H-M recruitment curve recorded at rest, prior to cycling.

##### Sensory stimulation

To induce sensory stimulation of the right foot, trains of 15× 1 ms pulses (50 Hz) were applied simultaneously to the superficial peroneal, tibial, and sural nerves with bipolar surface electrodes (see Figure 1C for electrode positions). Grass S88 stimulators (Grass Instruments, AstroMed Inc., West Warwick, RI, USA) connected in series with a SIU5 isolator and CCU1 constant current units were used to deliver the stimuli. The onset of sensory stimuli was triggered when the pedal passed the 2 o’clock position in the CONTROL + STIM and SPRINT + STIM conditions. Stimulation lasted for 10 s during the sprint (SPRINT + STIM) or at the same 10 s time point during unloaded cycling (CONTROL + STIM). Prior to the experimental protocol, perceptual threshold (PT) and radiating threshold (RT) was determined for each site. PT was defined as lowest current required to evoke the smallest detectable tactile sensation, whereas RT was defined as the minimum current required to cause clear radiating paresthesia of the innervation area (Nakajima et al., 2014). Stimulation intensity was set to 1.0× RT in order to induce a non-noxious cutaneous sensation. Participants described the sensation as “tingling”, “fluttering” and/or “vibrating”.

#### Electromyographic Recordings

Skin surfaces were shaved and then cleaned with alcohol wipes. Bipolar configurations of Ag-AgCl surface electrodes (Thought Technology Ltd., Montreal, QC, Canada) were fixed to the skin over muscles of the right SOL, TA, vastus lateralis, and flexor carpi radialis. Ground electrodes were fixed to bony landmarks (patella and lateral olecranon) that were electrically neutral. EMG recordings were amplified (500–1000 times for SOL and 5000 times for all other muscles) and filtered (10–1000 Hz for SOL and 100–300 Hz for all other muscles, P511 Grass Instruments, AstroMed Inc., West Warwick, RI, USA).

#### Cycle Ergometer Sprints

All cycling occurred on a Monark cycle ergometer (Ergomedic 894 E, Monark Exercise AB, Vansbro, Sweden). Unloaded cycling was maintained at 60 ± 5 rpm between sprints. For all sprints, participants were instructed to accelerate slowly to 60 rpm and then accelerate as hard as possible, at which point the weight basket (9% of body weight) would drop and they would cycle as hard as possible for 10 s. For all subsequent sprints, the participants were already cycling at 60 rpm (unloaded) and were given a 30 s warning and then a 3 s countdown of when they were to sprint again (see Figure 1A for timeline). No verbal encouragement was provided during the sprints, to maintain consistent external motivation between sessions. All power output data was recorded using Monark Wingate software and stored on a computer for further analysis.

### Data Analysis

All EMG data were acquired at a sampling rate of 5000 Hz with a 12-bit A/D converter connected to a personal computer running custom LabView version 8.0 (National Instruments, Austin, TX, USA). EMG data other than SOL were full wave rectified. SOL H-reflex and M-wave data were analyzed from single unrectified 100 ms sweeps. H-reflex and M-wave peak-to-peak amplitudes were analyzed in all trials with custom written Matlab version R2011b (Mathworks, Nantick, MA, USA) and then normalized to *M*_max_ amplitude. For recruitment curves, data was then imported into custom written LabView software where it was fit with a sigmoid function (Klimstra and Zehr, 2008). Pre-stimulus EMG activity was calculated as the root mean square value 20 ms prior to stimulus onset.

### Statistical Analysis

Mean power output was compared between conditions using a two-way repeated measures (RM) analysis of variance (ANOVA; 2 conditions (*SPRINT* vs. *SPRINT + STIM*) × 7 sprints). *M*_max_, H-reflex and corresponding M-wave amplitudes, and background EMG were compared between conditions with a two-way RM ANOVA (4 conditions × 10 time points). Current required to evoke H-reflex threshold, *H*_max_ amplitude, H-reflex @ 50% of current required to produce *H*_max_ and slope of the H-reflex recruitment curve at rest were compared from pre- to post-exercise with a two-way RM ANOVA (4 conditions × 2 time points; pre and post-exercise).

Pairwise comparisons were performed on significant main effects (condition and time) and interactions using paired *t*-tests. Data is expressed as means ± SD, except in figures where it is expressed as means ± SE for clarity. Significant differences were determined as *p* < 0.05 in all cases and all ANOVA and *t*-tests were performed using SPSS version 22 (SPSS, Chicago, IL, USA).

To provide qualitative information about effects of sensory stimulation, we included magnitude based-inferences. Effect sizes (Cohen’s *d*) on the interaction effects in the mean changes between conditions (*SPRINT + STIM* and *SPRINT*) were determined. The interaction effect of time and sensory stimulation was calculated from the mean difference between pre-exercise and each time point (post-sprint 1, 2, 3, 4, 5, 6, 7, post-5 mins and post-10 mins) for the *SPRINT + STIM* and *SPRINT* conditions. The two differences were then subtracted to estimate the effect of sensory stimulation at each time point. The following criteria were used to assess qualitative descriptors of standardized effects: trivial (<0.2), small (0.2–0.5), moderate (0.5–0.8) and large (>0.8; Cohen, 1988). Effects with 95% confidence limits that overlap the threshold for small positive and negative effects were defined as unclear. Effect sizes, which were clearly small or larger, were defined as substantial (Pearcey et al., 2015a). All magnitude-based inference calculations were performed in Excel version 2011 (Microsoft Corporation, Redmond, WA, USA).

## Results

### Power Output

#### Effects of Fatigue on Power Output

##### Sprint power is reduced with repeated bouts

**Figure 2:**
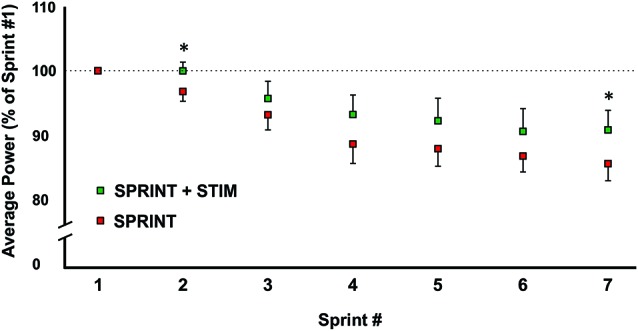
A graphical representation of how the average power output changed from sprints 1 through 7. All values were made relative to sprint 1 of the corresponding session. Asterisks indicate significant (*p* < 0.05) differences between *SPRINT + STIM* and *SPRINT* conditions. All values are means ± standard error.

The average power output of the first sprint between sessions was not significantly different (*SPRINT + STIM* = 745 ± 171.3 watts; *SPRINT* = 758 ± 171.5 watts; *p* = 0.84), but to reduce inter-session variability, each sprint was made relative to the first sprint of that session. For both the *SPRINT + STIM* and *SPRINT* conditions, as the sprint number increased, the mean power decreased (see Figure 2). The 2 (condition) × 7 (time) RM ANOVA revealed that there was a significant (main effect time, *p* < 0.001) decrease in average power output in during both sessions. There was a substantial small to very large effect (*d* ranged from 0.34 to 5.60) of sprint number on average power output (see Table 1).

**Table 1:** Effect sizes and 95% confidence intervals of between condition differences in percent change of average power from sprint 1 to each sprint (top) and effect sizes of decrease in average power from sprint 1 to each sprint within each condition (bottom).

% decrease in avg power		Sprint #					
		2	3	4	5	6	7
Cohen’s D (condition)		0.41	0.14	0.26	0.28	0.28	0.45
95% CI		0.24–3.90	−1.28–3.45	−0.08–5.17	−0.24–5.07	−0.35–4.84	1.23–6.29
Cohen’s D (time)	S	1.67	2.68	3.56	4.36	5.37	5.60
	S + S	0.34	1.87	2.50	2.49	2.93	3.26

#### Effects of Sensory Stimulation on Performance Measures

##### Sensory stimulation mitigates fatigue-related decline in power output

Overall, sensory stimulation resulted in smaller decrements of average power throughout the sprint protocol. The 2 (condition) × 7 (time) RM ANOVA revealed that there was a significant interaction effect (*p =* 0.034). Pairwise *t*-tests revealed that the decrease in average power was significantly greater for the *SPRINT* than *SPRINT + STIM* at sprint 2 (*p* = 0.048) and 7 (*p* = 0.036). Between-condition effects of sensory stimulation on power output are provided in Table 1.

### Spinal Cord Excitability

#### Spinal Cord Excitability Control Measures

##### Pre-exercise *M*_max_ and H-reflexes evoked during unloaded cycling were similar between conditions

**Figure 3:**
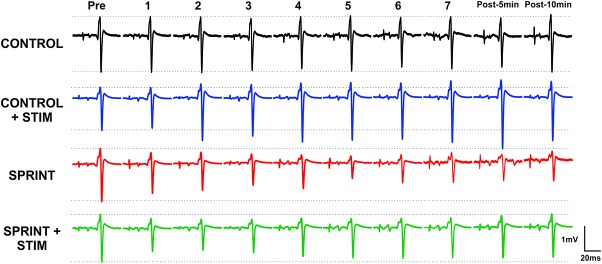
Depicts a single subject’s average of 10 H-reflex recordings at each time point, for each condition. Horizontal dotted lines indicate the pre-exercise H-reflex amplitude of each session for ease of comparison.

**Figure 4:**
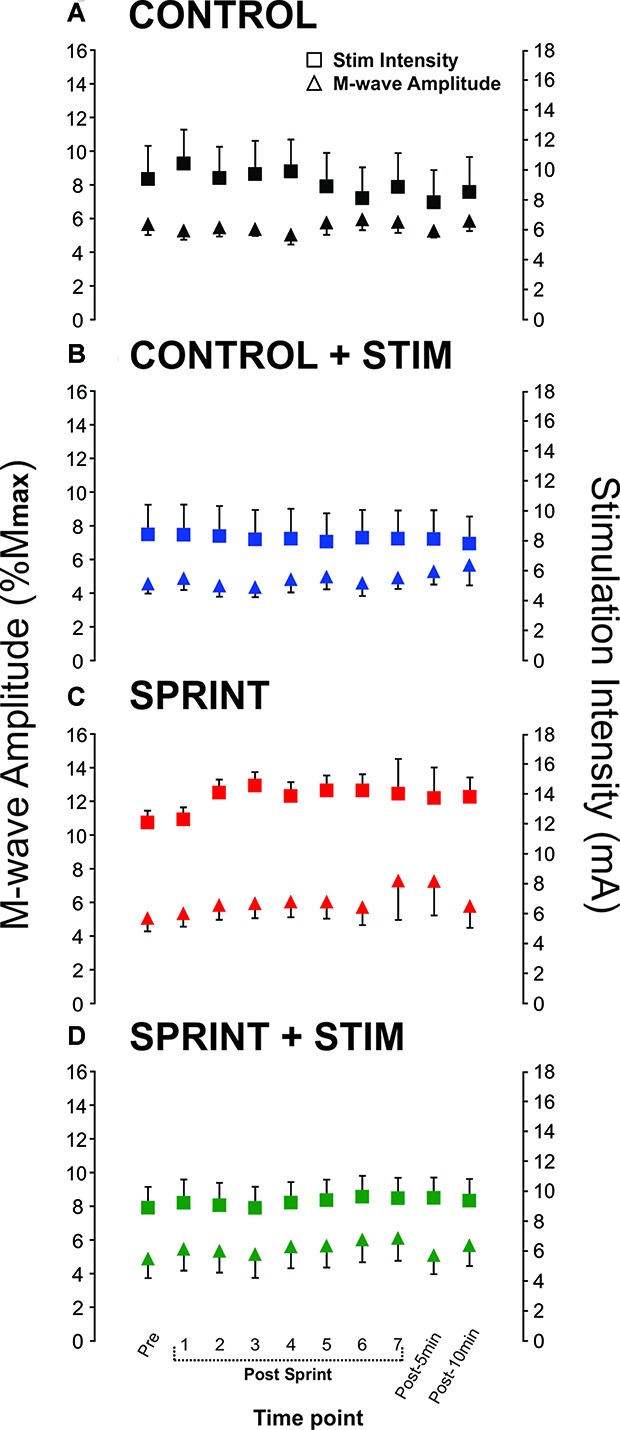
Group means of M-wave amplitudes that accompanied H-reflexes (primary *y*-axis) and stimulation intensities required to evoke H-reflexes (secondary *y*-axis) at each time point (*x*-axis) are represented by triangles and squares, respectively. Each panel displays data from the following condition: **(A)** *CONTROL*, **(B)** *CONTROL + STIM*, **(C)** *SPRINT* and **(D)** *STIM + SPRINT*.

**Figure 5:**
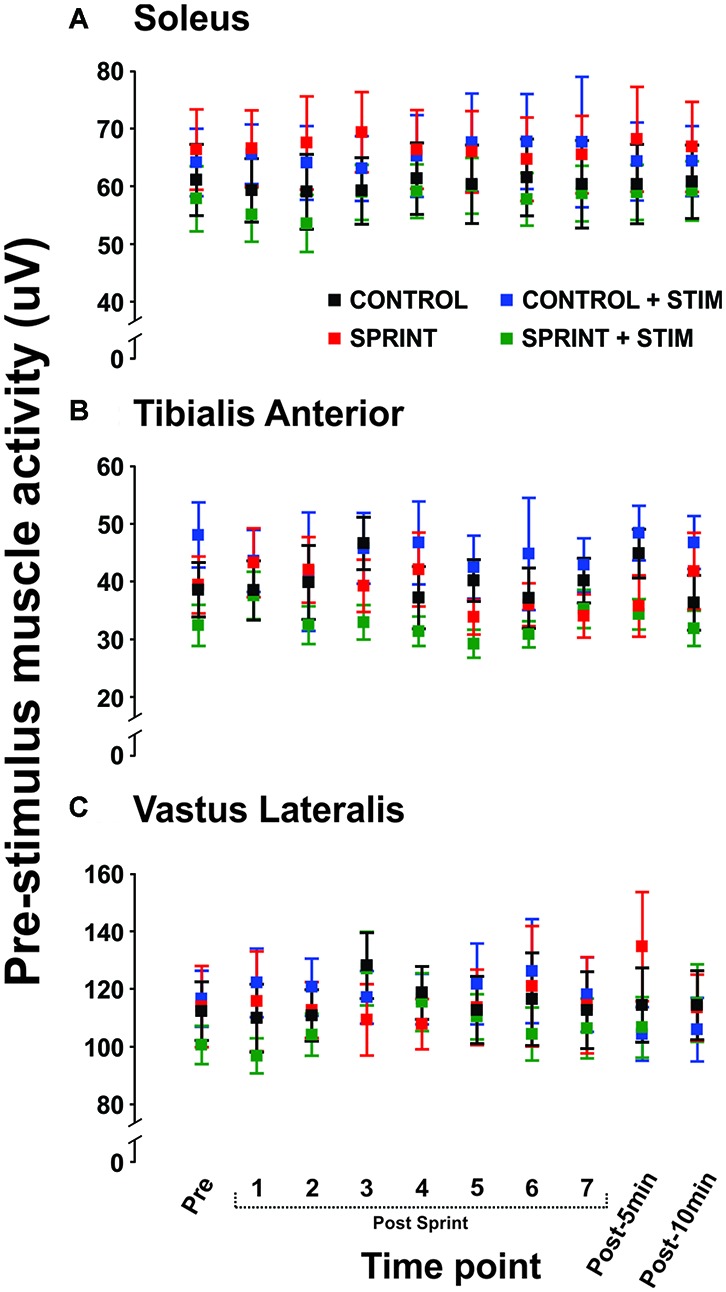
Group means of the rectified EMG amplitude averaged over 20 ms prior to stimulus onset is displayed for the soleus (**A**), tibialis anterior (TA; **B**), and vastus lateralis **(C)** for all time points that H-reflexes were evoked during unloaded cycling. In all panels, *CONTROL* is black, *CONTROL + STIM* is blue, *SPRINT* is red and *SPRINT + STIM* is green.

*M*_max_ amplitudes were not significantly (*p* = 0.61) different between or within conditions. Furthermore, H-reflexes were not significantly (*p* = 0.45) different pre-exercise between conditions, but to reduce inter-session variability, all H-reflex amplitudes were made relative to the pre-exercise value. H-reflex amplitudes evoked at pre in each condition as a percentage of both *H*_max_ and *M*_max_ are presented in Table 2. An exemplary subject’s H-reflex recordings for each condition and time point are shown in Figure 3. Furthermore, group mean stimulation intensity used to evoke H-reflexes and M-wave amplitudes recorded with H-reflexes at all time points for all conditions are shown in Figure 4. There were no significant effects observed for time (*p* = 0.91 [stim], *p* = 0.55 [M-wave]) or condition (*p* = 0.75 [stim], *p* = 0.32 [M-wave]), for both stimulation intensity and M-wave amplitude. To ensure that differences in muscle activity were not contributing to the differences in H-reflexes between time points and conditions, we plotted the group mean pre-stimulus EMG activity of the SOL, TA and VL in Figure 5. No significant time (*p* = 0.12 [SOL], 0.46 [TA], 0.67 [VL]) or condition (*p* = 0.42 [SOL], 0.72 [TA], 0.29 [VL]) effects were observed.

**Table 2:** Group averaged values for stimulation current, M-wave amplitude, H-reflex amplitudes as a percentage of *H*_max_ and H-reflex amplitudes as a percentage of *M*_max_ for H-reflexes evoked during unloaded cycling at PRE.

Condition	Stimulation current (mA)		M (% of *M*_max_)		H (% of *H*_max_)		H (% of *M*_max_)	
	Mean	SD	Mean	SD	Mean	SD	Mean	SD
CONTROL	8.4	7.16	4.6	2.13	77.4	13.65	40.8	16.73
CONTROL + STIM	9.6	8.23	5.1	2.85	75.9	22.71	38.0	15.94
SPRINT	8.9	6.05	5.4	4.52	77.1	19.68	43.5	22.24
SPRINT + STIM	9.4	8.06	5.3	2.18	75.8	21.73	41.4	20.60

##### Pre H-M recruitment curves were similar between conditions

**Figure 6:**
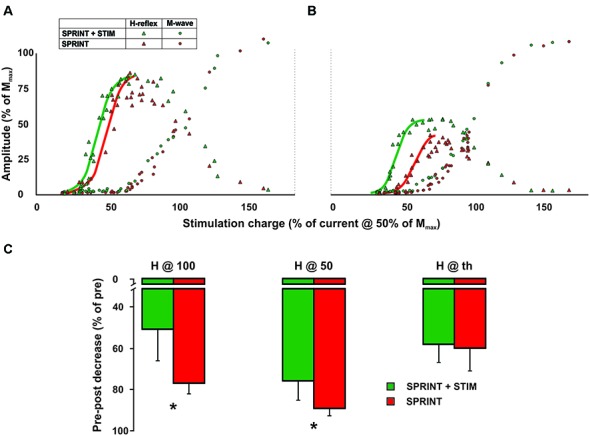
Single subject M-H recruitment curve raw data for the *SPRINT* (red) and *STIM + SPRINT* (green) conditions at **(A)** pre, and **(B)** post session. Triangles indicate H-reflex peak-to-peak amplitudes (*y*-axis), circles indicate M-wave amplitudes (*y*-axis) at the corresponding stimulation intensity (*x*-axis), solid red and green lines represent the sigmoid fit that was used to calculate the variables for the recruitment curve analysis similar to Klimstra and Zehr (2008). Group averages of pre to post session percent changes in recruitment curve variables are shown in **(C)**. These variables indicate the amplitude of the post H-reflex when measured at the same relative stimulation intensity that evoked a H-reflex at threshold (H@th), halfway up the ascending limb (H@50) and at the peak of the ascending limb (H@100). Please see Klimstra and Zehr (2008) for detailed descriptions of the methods used to determine these variables. Asterisks indicate significant (*p* < 0.05) differences in the decrease pre to post session between conditions. Values are group means ± standard error.

All parameters of the recruitment curves recorded at rest prior to the protocol were stable (e.g., were not statistically different; *p* = 0.63, 0.70, 0.83 for H@th, H@50 and H@100, respectively) between sessions. Figure 6A displays a single subject plot of the pre-exercise recruitment curves between the SPRINT and SPRINT + STIM conditions whereas Figure 6B displays the post-exercise recruitment curves between conditions.

#### Effects of Sensory Stimulation on Spinal Cord Excitability

##### Sensory stimulation facilitates H-reflexes evoked during control cycling

**Figure 7:**
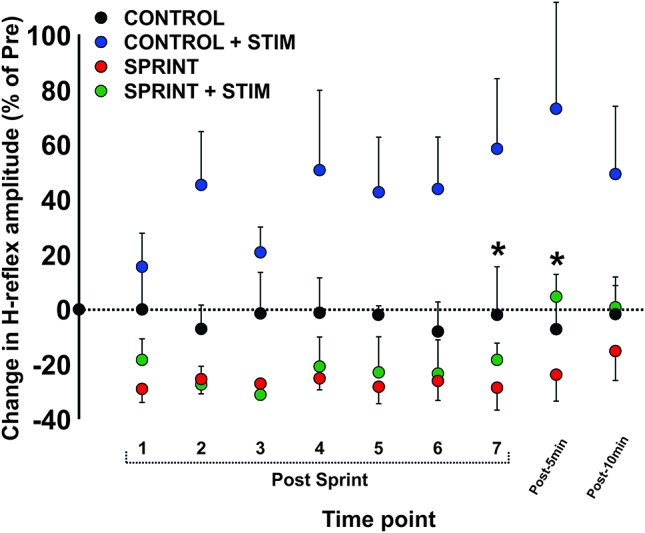
Depicts the group average H-reflex amplitudes relative to the *M*_max_ measured at the same time point. All values were made relative to the pre-exercise average of the corresponding session for comparison. Values are group means ± standard error. Asterisks indicate significant (*p* < 0.05) differences between *SPRINT + STIM* and *SPRINT* conditions. For clarity of display, all other significant differences were omitted and can be found in text.

During *CONTROL*, there was no amplitude modulation of H-reflexes over time (*p* = 0.23), however, sensory stimulation applied for 10 s caused a general increase of the H-reflex amplitudes throughout the *CONTROL + STIM* session. The 4 (condition) × 10 (time) RM ANOVA revealed that there was a significant (main effect condition, *p* = 0.016, main effect time, *p* = 0.008, interaction *p* = 0.035) difference in H-reflex amplitudes during the *CONTROL + STIM* condition. In fact, at all time points other than post 1 (*p* = 0.28), pairwise comparisons revealed that *CONTROL + STIM* H-reflex amplitudes were greater than all other conditions (*p* ranged from <0.001 to 0.03). The effects of sensory stimulation were substantially small to moderate (*d* ranged from 0.36 to 0.67) which resulted in increased H-reflex amplitudes throughout the experimental protocol (see Figures 3, 7; Table 3).

**Table 3:** Effect sizes and 95% confidence intervals of the between condition difference of the percent change of H-reflex amplitude from pre-sprint 1 to each time-point (top) and effect sizes of the change in H-reflex amplitude from pre-sprint 1 to each sprint within each condition (bottom).

Reflex amplitude % change		Time point								
	Comparison	Post-1	Post-2	Post-3	Post-4	Post-5	Post-6	Post-7	5 min post	10 min post
Cohen’s D (condition)	C + S − S	1.14	1.09	1.52	0.73	1.02	1.09	1.02	0.72	0.77
	C + S − S + S	0.98	1.14	1.64	0.59	0.77	0.88	0.86	0.47	0.50
	S − S + S	0.38	0.19	0.20	1.01	0.83	0.56	0.92	1.00	0.62
	C − C + S	−0.16	−1.55	−0.38	−1.26	−5.08	−1.52	−0.94	−1.77	−1.03
	C − S + S	0.39	0.61	0.53	0.41	2.02	0.37	0.27	−0.24	−0.01
	C − S	0.59	0.62	0.50	0.63	2.91	0.51	0.46	0.37	0.21
95% CI (condition)	C + S − S	35.00–43.06	55.03–64.77	35.99–43.63	54.93–66.22	53.17–63.18	53.73–63.72	68.23–78.95	72.21–85.83	47.81–59.47
	C + S − S + S	29.41–38.12	56.81–67.77	38.61–43.63	41.38–54.94	37.03–49.35	40.91–52.92	55.53–67.67	43.86–58.56	28.06–40.61
	S − S + S	1.77–8.73	1.39–6.25	1.76–6.63	−0.35–8.04	−0.30–10.17	−0.36–6.96	−0.81–8.24	0.24–23.04	0.14–14.35
	C −C+S	−11.6 to −3.14	−49.50 to −40.29	−22.62 to −15.45	−58.84 to −47.96	−52.42 to −43.90	−59.02 to −50.10	−60.34 to −49.63	−79.77 to −67.30	−55.95 to −45.65
	C − S + S	15.02–21.84	14.50–20.72	23.46–29.90	14.49–20.20	16.73–21.59	10.26–16.40	12.25–18.85	−13.32 to −6.41	−4.33–2.91
	C − S	24.67–30.83	15.30–20.77	21.96–28.37	24.06–29.90	25.16–30.13	14.96–21.31	23.74–29.88	12.27–18.72	7.03–14.12
Cohen’s D (time)	C+S	0.36	0.67	0.57	0.46	0.58	0.65	0.65	0.52	0.55
	S	1.92	1.55	1.47	1.92	1.47	1.19	2.12	0.80	0.49
	S + S	0.99	1.39	1.76	0.35	0.30	0.36	0.81	0.24	0.14
	C	−0.02	0.25	0.03	0.03	0.18	0.23	0.04	0.18	0.06

*C = CONTROL, C + S = CONTROL + STIM, S = SPRINT, S + S = SPRINT + STIM*.

#### Effects of Fatigue on Spinal Cord Excitability

##### H-reflexes evoked during unloaded cycling are suppressed following intermittent sprints

There was general suppression of H-reflexes evoked after each of the seven sprints for both the *SPRINT* and *SPRINT + STIM* conditions, when compared to pre-exercise. A 4 (condition) × 10 (time) RM ANOVA revealed that there was a significant (main effect condition, *p* = 0.016, main effect time, *p* = 0.008, interaction *p* = 0.035) change in H-reflex amplitude during the *SPRINT + STIM* and *SPRINT* conditions. Pairwise comparisons revealed that *SPRINT* (all *p* < 0.001) and* SPRINT + STIM* (*p* ranged from < 0.001 to 0.022) H-reflex amplitudes were reduced after sprints 1 through 7. There were both trivial and substantial small to large (*d* ranged from 0.14 to 1.92) effects of intermittent sprints to reduce H-reflex amplitudes (see Table 3). Interestingly, there was no longer significant suppression of H-reflex amplitudes measured at 5 (*p* = 0.38) or 10 min (*p* = 0.39) post sprint 7 for the *SPRINT + STIM* condition, whereas the suppression remained at 5 (*p* = 0.005) and 10 min (*p* = 0.036) post-sprint 7 for the *SPRINT* condition.

#### Interaction of Sensory Stimulation and Fatigue on Spinal Excitability

##### Sensory stimulation mitigates fatigue-related suppression of H-reflexes measured during unloaded cycling following intermittent sprints

This is evidenced by a reduction of H-reflex suppression in the *SPRINT + STIM* compared to *SPRINT* condition. A 4 (condition) × 10 (time) RM ANOVA revealed that there were significant (main effect condition, *p* = 0.016, main effect time, *p* = 0.008, interaction effect, *p* = 0.035) differences between SPRINT and SPRINT + STIM. Paired *t*-tests revealed that the *SPRINT + STIM* H-reflexes were significantly less suppressed immediately (*p* = 0.006) and 5 mins after sprint 7 (*p* = 0.019) compared to the *SPRINT* condition (see Figures 3, 7; Table 3).

##### Sensory stimulation mitigates fatigue-related suppression of resting H-M recruitment curves measured post-exercise

A 4 (condition) × 2 (time) RM ANOVA for each variable revealed that from pre- to post-experimental protocol, there were significant main effects for time that indicated an increase in the slope of the H recruitment curve (*p* < 0.001) and current required to evoke 50% of *H*_max_ (*p* = 0.003) and *H*_max_ (*p* = 0.018) and, furthermore, a decrease in the post H-reflex amplitude evoked at 50% of the current required to get *H*_max_ during the pre-exercise test (*p* < 0.001) and the post H-reflex amplitude evoked at the current required to evoke the pre-exercise *H*_max_ (*p* < 0.001). Main condition effects were observed for the H-reflex amplitude evoked at the current used to evoke *H*_max_ during the pre recruitment curve (*p* = 0.034) and H-reflex amplitude evoked at 50% of current required to evoke *H*_max_ during the pre recruitment curve (*p* = 0.045). Pairwise *t*-tests revealed that both of these variables were greater for the *SPRINT* condition compared to the *SPRINT + STIM* condition (see Figure 6C).

## Discussion

The main results from this experiment show that sensory stimulation can alter the effects of fatigue; furthermore spinal cord excitability is reduced by fatigue and enhanced by sensory stimulation. These data reveal for the first time the potential effects of cutaneous sensory input interacting with fatigue-induced suppression of neural function and performance outcomes. These observations further our understandings of the effects of cutaneous input on human locomotion. Potential mechanisms and methodological considerations are discussed below.

### Sensory Stimulation Reduces Decrements in Performance Measures

With increasing bouts of intermittent sprints, power output decreased significantly. This is a consistent finding with previous work that has examined the fatigue of intermittent sprints during arm (Pearcey et al., 2016) and leg cycling (Billaut et al., 2006; Billaut and Basset, 2007; Racinais et al., 2007a; Mendez-Villanueva et al., 2008; Girard et al., 2013a,b; Pearcey et al., 2015b). A noteworthy finding of the current experiment was that there was significantly less decrement in average power output of the second and seventh sprints in the presence of sensory stimulation compared to sprints without stimulation. Although the mechanism is not entirely clear, it is possible that information from distal afferent nerve stimulation interferes with group III/IV afferent information, which has been speculated to contribute to fatigue during intense exercise (Amann et al., 2008, 2011; Sidhu et al., 2014, 2017). Since stimulation of the distal tibial, sural and superficial peroneal nerves can activate multiple sensory afferents, trains of stimuli applied during each pedal stoke may act to send volleys of action potentials to the spinal cord and supraspinal centers. Although it is unclear if enhanced sensory information may interact with the perception of difficulty resulting from an intense bout of exercise, it appears that there are slight interactions of peripheral nerve stimulation with repeated sprint ability. Further work is needed to understand the exact mechanisms contributing to these effects.

### Sensory Stimulation Facilitates Spinal Cord Excitability During Unloaded Cycling

During unloaded control cycling, we stimulated the right foot each time the foot passed the 2 o’clock position for a total of 10 pedal strokes, interleaved by 3 min of unloaded cycling. This stimulation facilitated the H-reflex amplitude by ~7%–27% of pre-session values throughout the experimental protocol. The amount of facilitation appeared to be least after the first bout of stimuli and greatest 5 min following the final bout of stimuli. This relationship suggests that, perhaps, there was a cumulative effect of sensory stimulation on the facilitation of the SOL H-reflex. We attribute the facilitation of the H-reflex to result from reduced Ia presynaptic inhibition. In the cat, Brink et al. (1984) showed that stimulation of group I afferents has premotoneuronal effects on Ia reflex excitability, such that stimulation can reduce presynaptic inhibition and, therefore, increase Ia reflex excitability. Low intensity stimulation of the sural nerve increases H-reflex amplitudes at C-T intervals of 70–90 ms (Demairé and Ciancia, 2000) and when stimulation of sural and common peroneal nerves are combined with condition-test intervals of 70–250 ms, there is significant decreases in presynaptic inhibition (Iles, 1996). Thus, it is apparent that group I afferents affect the excitability of the H-reflex. In the current experiment, a low intensity combination of sural, tibial and superficial peroneal stimulation of the foot resulted in facilitation of H-reflexes that were measured ~10–30 s following the sensory stimulation. Although a persistent effect of sensory stimulation has not been observed before, we suggest that there is a cumulative effect of the stimulation that can interact with the voluntary output during cycling, which acts to reduce presynaptic inhibition and therefore facilitate the SOL H-reflex.

### Fatigue Reduces Spinal Cord Excitability

Intermittent sprints induced suppression of SOL H-reflex amplitude. During non-fatiguing cycling, it has been speculated that suppression of the H-reflex occurs via Ia presynaptic inhibition (Brooke et al., 1997; Frigon et al., 2004) that likely arises from central pattern generator and/or descending supraspinal inputs. Therefore, since intermittent sprints and the subsequent rest periods in this experiment consisted of rhythmic cycling, increased presynaptic inhibition should be noted as a contributing factor to the suppression of the H-reflexes. Furthermore, electrically-stimulated submaximal fatiguing isometric contractions (Garland and McComas, 1990), sustained maximal voluntary isometric contractions (MVCs; Duchateau and Hainaut, 1993), sustained 25 and 50% MVCs (Duchateau et al., 2002), sustained low (25% MVC) and high (42%–66% MVC) force plantarflexion (Kuchinad et al., 2004) and intermittent MVCs (Iguchi and Shields, 2012) all cause approximately ~40%–50% reduction in H-reflex peak-to-peak amplitudes. In the current experiment, the H-reflex amplitudes were only decreased by ~11%–27%. Since the fatigability of the plantarflexors is not as substantial as some other muscle groups (i.e., knee flexors) during cycling sprints (Rampinini et al., 2016), it is possible that the SOL in the current experiment was not fatigued to the extent of the aforementioned isometric fatigue studies (Garland and McComas, 1990; Duchateau and Hainaut, 1993; Duchateau et al., 2002; Kuchinad et al., 2004). However, unlike the amount of amplitude modulation, the timeline of recovery of the H-reflex amplitude in the current experiment was perhaps longer than that of isometric contraction experiments. H-reflex amplitudes recorded 5–10 mins following the final sprint remained lower than values recorded prior to sprint 1 (see Figure 7) for the *SPRINT* condition. This trend was altered in the SPRINT + STIM condition, such that H-reflex amplitudes were back to pre-sprint values <5 mins following sprint 7. This recovery is similar to the recovery of H-reflex amplitudes seen in the work of Kuchinad et al. (2004). In the current experiment, the fatigue-related suppression after each sprint was quite similar, and did not increase with an increased number of sprints (i.e., more fatigue). The onset of suppression of H-reflexes in isometric contractions is early, and this suppression remains somewhat consistent throughout fatigue protocols (Duchateau et al., 2002; Kuchinad et al., 2004; Iguchi and Shields, 2012). These data indicate that the overall suppression of H-reflexes may arise from initial accumulation of metabolites in the muscles, which then activate group III/IV muscle afferents, similar to fatigue during locomotor exercise (Blain et al., 2016). Fatigue sensitive afferents can project to the spinal cord to inhibit extensor motoneuron output directly (Martin et al., 2006). It has also been shown that capsaicin sensitive group III/IV muscle afferents mediate inhibition of Ia input onto motoneurons (Pettorossi et al., 1999) through either presynaptic inhibitory interneurons or through direct synaptic connections onto group Ia afferent terminals (Della Torre et al., 1996). Fatigue sensitive afferents also project to supraspinal regions (Sidhu et al., 2017) and likely act to alter supraspinal excitability of the corticospinal tract (Pearcey et al., 2016), which can alter descending commands on presynaptic inhibition.

Although peripheral changes in excitability (i.e., sarcolemma excitability) can contribute to a reduction in H-reflex amplitude, these changes would be evident by a decrease in the *M*_max_ amplitude. Since *M*_max_ amplitudes did not significantly decrease, but H/*M*_max_ did decrease throughout the SPRINT protocol, it can be concluded that these changes were not due to a decrease in peripheral excitability. The lack of a decrease in *M*_max_ amplitude throughout a sprint protocol is similar to that of the *M*_max_ amplitudes evoked in the knee flexors in previous studies (Billaut et al., 2013; Hureau et al., 2014; Pearcey et al., 2015b). Decreased excitability of Ia afferent axonal excitability (hyperpolarization) can also contribute to the suppression of H-reflex amplitudes. If this were the case in the current experiment, one would expect to see an increase in current required to elicit H-reflexes, which would indicate an alteration in the axonal thresholds. There were no significant differences in stimulation intensity after each sprint or in pre- to post-session current required to evoke H-reflexes during the M-H recruitment curves. Thus, decreased excitability of Ia afferent axons likely is not responsible for the suppression of H-reflexes in the current experiment.

### Sensory Stimulation Interacts with Fatigue-related Reductions in Spinal Cord Excitability

H-reflex amplitudes immediately and 5 mins after sprint 7 were significantly greater in the *SPRINT + STIM* condition compared to the *SPRINT* condition. This suggests that the presynaptic inhibition of the *SPRINT + STIM* condition was slightly less than the *SPRINT* condition. Above, we have discussed how the intermittent sprints increase presynaptic inhibition of the Ia terminals on SOL motoneurons and that sensory stimulation during unloaded cycling reduces said presynaptic inhibition. Furthermore, we pointed out that there seems to be a cumulative effect of sensory stimulation that results in a greater facilitation of reflexes later in the session, whereas the increased presynaptic inhibition resulting from fatigue appears to be similar throughout the session. Therefore, the interactions of the effects of sensory stimulation and fatigue would likely be greatest later in the session. This is supported by a similar suppression of H-reflex amplitude between the *SPRINT + STIM* and *SPRINT* conditions early in the session, whereas later in the session, there is a divergence between the relative amplitudes of reflexes between the two conditions. Since, reductions in afferent input can lower firing rates of motoneurons (Macefield et al., 1993) and reduced presynaptic inhibition (i.e., increased afferent input to motoneurons) has been linked to the ability to maintain sustained isometric contractions (Baudry et al., 2011), it then seems possible that the reductions in group Ia presynaptic inhibition may have contributed to the improved performance of the cycling sprints.

## Conclusion

The novel interaction of stimulating nerves innervating the skin of the foot with the fatigue-related decrements in power and suppression of H-reflexes has been highlighted in the current study. This suggests that enhancing feedback from the skin may moderate exercise-related fatigue. The interactions between enhanced sensory input and fatiguing exercise implies that other modalities that alter the sensory transmission from the skin during fatiguing exercise may also have beneficial effects. Future experiments should be conducted to determine whether accessories, apparel or footwear, which can alter sensory feedback from the skin, are able to interact with exercise-related fatigue.

## Author Contributions

GEPP, BM and EPZ were involved with the conception of the experimental protocol, GEPP and SAN collected and analyzed the data, GEPP and EPZ interpreted the data, GEPP drafted the manuscript, GEPP, SAN, BM and EPZ revised the manuscript and have approved and are accountable for all aspects of the work.

## Conflict of Interest Statement

The authors declare that the research was conducted in the absence of any commercial or financial relationships that could be construed as a potential conflict of interest.
